# Hip Fractures in Malta: Are we Missing an Opportunity?

**DOI:** 10.1055/s-0041-1731635

**Published:** 2021-07-22

**Authors:** Mark Bugeja, Arthur Curmi, Daniel Desira, Gregory Apap Bologna, Francesco Galea, Ivan Esposito

**Affiliations:** 1 Department of Trauma and Orthopaedics, Mater Dei Hospital, Malta

**Keywords:** hip fractures, fragility fractures, calcium, vitamin D, bisphosphonates

## Abstract

**Introduction** Osteoporosis is a bone disease that is both preventable and treatable. It usually becomes evident when a fragility fracture occurs. Unfortunately, most studies show that only a small percentage of individuals at increased risk of fracture are assessed and treated, even following a fragility fracture.

**Objective** The aim of this study was to determine whether patients suffering from a low-energy hip fractures in the Maltese Islands are given osteoporosis treatment.

**Method** All patients older than 50 years presenting to the acute care hospitals in Malta and Gozo with a fragility hip fracture during December 1, 2015 and November 30, 2016 were included. Data on mortality, other fragility fractures, prescription of calcium, vitamin D, and antiresorptive therapy were collected.

**Results** Calcium with vitamin D supplements were prescribed to 40% of patients; however, only 2.64% of patients were given pharmacological therapy. Following a hip fracture, the mortality rate was 18.5% at 1 year and 26.21% at 2 years. Apart from a high mortality rate, 28.19% of individuals sustained another fragility fracture before or after the hip fracture.

**Conclusion** There should be increased osteoporosis awareness in Malta and a national bone mineral density screening program should be set up. An active role of the orthogeriatrics team in the management and treatment of osteoporosis following a fragility fracture might improve treatment rate and decrease refracture and mortality rates.

At the consensus development conference in 1990, osteoporosis was defined as “ *a disease characterized by low bone mass, microarchitectural deterioration of bone tissue leading to enhanced bone fragility, and a consequent increase in fracture risk* .” 1 It is referred to as a “silent disease,” as it is usually asymptomatic, and becomes evident when a fragility fracture occurs. 2 3 4 5 Hence, the clinical importance of this condition rests on the fragility fractures that arise. 6 These fragility fractures occur in the lumbosacral spine, distal radius, hip, and proximal humerus. Since bone mineral density (BMD) is low, the risk of fractures at other sites is also increased. 6 Of these, hip fractures are the most serious, causing great social and economic burdens, and leading to increased morbidity, mortality, and disability, with most of these patients becoming institutionalized. 7 8 9 10 11 12 Melton 13 estimated the lifetime risk of hip fracture at 17.5 and 6% in 50-year-old white women and men, respectively ( Table 1 ). The risk is comparable to the other major fragility fractures involving the spine and wrist. 13 Following an osteoporotic fracture, one is at an increased risk for further fragility fractures. 14 15 To prevent this, several guidelines for the diagnosis and treatment of osteoporosis were developed. However, its management remains a challenge, especially in the management of secondary causes, effectiveness and treatment period of antiresorptive agents, and the role of calcium and vitamin D supplements. 16

**Table 1:** Estimated lifetime fracture risk in 50-year-old white women and men

	Women % (95% CI)	Men % (95% CI)
Proximal femur fracture	17.5 (16.8, 18.2)	6.0 (5.6, 6.5)
Vertebral fracture	15.6 14.8, 16.3)	5.0 (4.6, 5.4)
Distal forearm fracture	16.0 (15.2, 16.7)	2.5 (2.2, 3.1)
Any of the three	39.7 (38.7, 40.6)	13.1 (12.4, 13.7)

Abbreviation: CI, confidence interval.

*Source* : Table taken from Melton. 13

## Detection of Disease

In 1994, the World Health Organization (WHO) study group described osteoporosis in white postmenopausal women as a BMD value of 2.5 standard deviations (SD) or more below the young adult mean (T-score ≤ −2.5). In the presence of one or more fragility fractures and a BMD value more than 2.5 SD below the young adult mean, it is defined as severe osteoporosis. 17 For these diagnostic categories, BMD is converted into a T-score, which indicates the number of SD above or below the mean in healthy young adults ( Table 2 ). 18 A major problem with BMD measurement is that, alone it is inadequate for the detection of individuals at high risk of fracture, with tests having high specificity but low sensitivity. 19 Undeniably, most fragility fractures occur with a negative test. 19

**Table 2:** WHO four general diagnostic categories in osteoporosis 17 18

Diagnostic categories	BMD T-score
Normal	−1.0 or above
Low bone mass (osteopenia)	Between −1.0 and −2.5
Osteoporosis	−2.5 or below
Severe osteoporosis (established osteoporosis)	Below −2.5 in the presence of one or more fragility fractures

Abbreviations: BMD, bone mineral density; WHO, World Health Organization.

X-ray absorptiometry is the most commonly used technique for measuring BMD. This utilizes the high sensitivity of calcium in absorbing X-rays to measure the relative amount in bones and soft tissue, hence, calculating bone mineral content and density. 20 BMD at the hip is the most accurate for predicting risk of hip fractures, with spinal BMD used for monitoring treatment. This is because measurements at the lumbar spine can be heavily influenced by artifacts. 20 In cases where the hip or spine cannot be measured or interpreted, the forearm is used instead; however, there can be significant differences in BMD between the dominant and nondominant arm. 20

## Epidemiological Risk Factors

There are other risk factors other than BMD that predispose to fragility fractures. These include age, sex, diseases, and medications affecting bone strength, prior fragility fracture, family history of fragility fracture, body mass index, history of falls, smoking, alcohol, and physical inactivity. 16 19 Peak bone mass occurs by the age of 30 but this decreases steadily after 40 years of age and continues at an accelerated rate around the menopausal years before slowing down again after the age 70. 21 Age is therefore the strongest predictor for fracture risk. The FRAX ( www.shef.ac.uk/FRAX/ ) and QFracture ( www.qfracture.org ) are two major scores based on risk factors and used to estimate the 10-year probability of fracture. 16

## Guidelines

The American Association of Clinical Endocrinologists and American College of Endocrinology in 2016 published clinical practice guidelines on the diagnosis and treatment of postmenopausal osteoporosis ( Table 3 ). 22 One of the criteria stated, that in the presence of a low energy spine or hip fracture, regardless of BMD, patients are at a high risk for further fractures, thus, should be diagnosed with osteoporosis and considered for treatment. 22 The European Society of Endocrinology recommends pharmacological treatment to be started without a delay in patients with recent fractures to prevent further fragility fractures. 23 Both the National Osteoporosis Foundation (NOF) and the UK National Osteoporosis Guideline Group (NOFF) recommend starting treatment following a fragility fracture. 4 24

**Table 3:** 2016 AACE/ACE diagnostic criteria for osteoporosis in postmenopausal women

1	T-score −2.5 or below in the lumbar spine, femoral neck, total, and/or 33% radius
2	Low-trauma spine or hip fracture (regardless of BMD)
3	Osteopenia or low bone mass (T-score between −1 and −2.5) with a fragility fracture of proximal humerus, pelvis, or possibly distal forearm
4	Low bone mass or osteopenia and high FRAX fracture probability based on country-specific thresholds

Abbreviations: AACE/ACE, American Association of Clinical Endocrinologists/American College of Endocrinology; BMD, bone mineral density.

Source: Taken from Camacho et al. 22

## Treatment Options

As an adjunct to the osteoporosis therapies mentioned below, patients should be counseled on adequate calcium and vitamin D intake, fall prevention strategies, stopping smoking, limiting alcohol intake, and regular physical activity. 22 23

## Calcium

Lifelong sufficient intake of calcium is needed to achieve peak bone mass and later maintenance of bone health. When intake is inadequate, in an attempt to maintain serum calcium, the body resorbs bone, decreasing the BMD. According to NOF men between the age of 50 and 70 years should consume 1000 mg of calcium daily, while women older than 50 and men older than 70 should consume 1200 mg of calcium daily. 4 The most commonly used calcium supplements are calcium citrate and calcium carbonate, both of which are equally well absorbed when taken with food. 25

## Vitamin D

Vitamin D has a key role in calcium homeostasis. According to the NOF, the daily intake of vitamin D for those older than 49 years is between 800 and 1,000 international units (IU). 4 Unfortunately, 90% of those aged between 51 and 79 years have inadequate intake of vitamin D. 25 In view of variations in vitamin D requirements between individuals, the dose of vitamin D supplements should be titrated according to serum 25(OH)D levels. In fact, many osteoporotic patients require more than what the NOF recommends. 4 Vitamin D deficiency may be treated with 7,000 IU vitamin D _2_ or vitamin D _3_ for 8 to 12 weeks to attain a serum 25(OH)D level of around 30 ng/mL. 4 This is followed by maintenance dose (usually 1,500–2,000 IU daily) adjusted according to target serum 23(OH)D level. 4

## Bisphosphonates

Bisphosphonates are stable analogues of pyrophosphate with powerful effects on bone metabolism. 26 They preferentially bind to the surface of bone at locations of active remodeling. In the acidic environment of the bone resorption sites, bisphosphonates detach form the bone mineral, attach to osteoclasts, and impair their function. 26 Bisphosphonates may cause renal impairment and very rarely may cause osteonecrosis of the jaw. 4 Long-term use of bisphosphonates is associated with low-energy atypical femur fractures. 4 Thus, fracture risk should be recalculated after 3 to 5 years, with women who remain at high risk of fractures continuing treatment, while those having low-to-moderate risk of fractures being considered for a “bisphosphonate holiday” (temporary cessation of bisphosphonate for up to 5 years). 23

## Denosumab

Denosumab is a human monoclonal antibody against the receptor activator of nuclear factor kappa-β ligand, thus, interfering with the formation, activation, and survival of osteoclasts. 5 24 27 Denosumab is given subcutaneously every 6 months. Patients on denosumab can develop hypocalcemia; thus, sufficient calcium and vitamin D intake is of paramount importance, especially in those with severe kidney impairment. 24 Also, serum calcium levels should be checked before every dose of denosumab and within 2 weeks after initial dose. 24 Similar to bisphosphonates, denosumab can also cause osteonecrosis of the jaw and atypical femur fractures. 24 Patients on denosumab should have fracture risk recalculated after 5 to 10 years, and women who remain at high risk of fractures either continue or else change therapy. 23

## Teriparatide and Abaloparatide

Teriparatide is parathyroid hormone (PTH), while abaloparatide is a PTH-related protein analog. 23 These are the two licensed peptides that are anabolic for bone (i.e., increase bone formation) when administered intermittently. 23 Both agents may cause an increase in serum calcium levels; thus, serum calcium level should be checked before starting treatment. 23 The European Society of Endocrinology recommends treatment with these agents for 2 years, then continue with antiresorptive medication to maintain the BMD gains. 23

## Selective Estrogen Receptor Modulators

Selective estrogen receptor modulators (e.g., raloxifene) are agents that interact with intracellular estrogen receptors in target organs as estrogen agonists and antagonists. 28 When compared with menopausal hormone therapy, it has less impact on BMD. 23 They are indicated for postmenopausal women with osteoporosis in whom bisphosphonates or denosumab are not appropriate and who have low risk of thrombosis and high risk of breast cancer. 22 23

## Menopausal Hormone Therapy

According to the European Society of Endocrinology, menopausal hormone therapy should only be considered in women younger than 60 years or who are less than 10 years following menopause in whom bisphosphonates or denosumab are not indicated. 23 Patients should not have any history of myocardial infarction, cerebrovascular accidents, breast cancer, or risk of thrombosis. 23 Estrogen on its own should only be used in women with hysterectomy. 22 23

## Calcitonin

Calcitonin should only be used in patients in whom the above-mentioned therapies are not appropriate. 4 22 23 It is administered either as nasal spray (200 IU daily) or injected subcutaneously or intramuscularly (100 IU daily). Skin testing is recommended before using calcitonin to avoid hypersensitivity. 22 In view of concerns about increased risk of cancer especially liver and prostate, the European Medicines Agency and Health Canada have withdrawn the nasal spray from the market. 23

## Objective of the Study

Hip fractures in Malta are managed using a multidisciplinary team; unfortunately, orthopaedic surgeons often rely on primary care physicians to take responsibility for the management of osteoporosis. 29 Since these fractures are usually admitted, orthopaedic surgeons are the first contact for these patients, and this gives them the opportunity not only to treat the fracture but also to start osteoporosis treatment. 3 14 The objective of this observational study is to determine whether patients sustaining low-energy hip fracture in the Maltese Islands were given osteoporosis treatment.

## Method

All individuals older than 50 years presenting to the acute care hospitals in Malta and Gozo with low-trauma hip fractures during December 1, 2015 and November 30, 2016 were included in this study. They were identified retrospectively using the hospitals' imaging software, Picture Archiving and Communication System. Using the same software, prior (10 years before) and subsequent fragility fractures of the hip, distal radius, proximal humerus, and spine were recorded. Using the hospitals' electronic case summary software, the list of medications on discharge from both the acute hospitals and the rehabilitation hospital was obtained. From the drug history on the discharge summary, those prescribed calcium, vitamin D, and antiresorptive therapy were noted. Patients' demographics, date deceased, and length of hospital stay were obtained using the hospitals' i. Clinical Manager (i.CM) version 2.1. The in-hospital, 1-year, and 2-year mortality following the hip fracture was calculated.

## Results

There were 454 individuals older than 50 years who suffered from a low-energy hip fracture between December 1, 2015 and November 30, 2016. The average age at presentation was 80.3 years with two-thirds (72.7%; *n* = 330) being women. Calcium and vitamin D supplements were given to 40% of individuals ( *n* = 182). Calcium supplements on their own were given to 5% of patients ( *n* = 23). The majority (55%; *n* = 249) were not given any supplements ( Table 4 ).

**Table 4:** Calcium, vitamin D supplements, and antiresorptive medications

	Women ( *n* = 330)	Men ( *n* = 124)	Total	Percentage
Calcium and vitamin D	143	39	182	40
Calcium only	15	8	23	5
No supplements	172	77	249	55
Antiresorptive medications	10	2	12	2.64

Only 12 patients (2.64%) were given osteoporosis therapy ( Table 4 ). Most of these patients ( *n* = 9) were prescribed therapy from a rehabilitation hospital, two patients were already on treatment when they suffered from a hip fracture, while only one patient was prescribed treatment form the acute hospital. Of the osteoporosis treatments, bisphosphonates were the most prescribed, and only one patient received the monoclonal antibody denosumab. The bisphosphonate of choice was alendronic acid. This was given to eight patients. The other three patients receiving bisphosphonates were given zoledronic acid, risedronate sodium, and ibandronic acid.

The in-hospital mortality was 5.95% ( *n* = 27), which rose to 18.50% ( *n* = 84) by 1 year, and 26.21% ( *n* = 119) after 2 years following the hip fracture. Apart from having an increased risk of mortality, more than one in four patients ( *n* = 128; 28.19%) suffered from a fragility fracture before or subsequent to the hip fracture ( Table 5 ). Thirty-nine patients (8.59%) suffered from another hip fracture, while the same number of patients ( *n* = 39) suffered from a fracture of the distal radius. Thirty-seven patients (8.15%) suffered from a proximal humerus fracture, while spine fractures were present in only 13 patients (2.86%).

**Table 5:** Fragility fractures prior or subsequent to the hip fracture

Prior or subsequent fragility fracture	No. of individuals (%)
Contralateral hip fracture	39 (8.59)
Fracture of the distal radius	39 (8.59)
Proximal humerus fracture	37 (8.15)
Spinal fracture	13 (2.86)
Total	128 (28.19)

Most patients suffering from hip fractures are elderly with multiple comobilities taking several medications. Polypharmacy is considered a crucial risk factor for falls in older people, with the risk of falls increasing with the number of medicines taken per day. 30 Certain medicines are associated with increased risk of falls. These include antihypertensives, diuretics, antipsychotics, and antidepressants. 30 On the other hand, there are medications that decrease the BMD. These include proton pump inhibitors, glucocorticoids, anticoagulants, insulin, and oral hypoglycemic agents. 31 Table 6 shows the frequency of these commonly prescribed medicines.

**Table 6:** Commonly prescribed drugs that may increase the risk of falls or decrease bone mineral density

Type of drug	No. of individuals taking these medications (%)
Antihypertensives	247 (54.41)
Diuretics	174 (38.33)
Antidepressants	73 (16.08)
Antipsychotics	51 (11.23)
Proton pump inhibitors	157 (34.58)
Glucocorticoids	20 (4.41)
Anticoagulants	108 (23.79)
Antiplatelets	126 (27.75)
Oral hypoglycemic agents	103 (22.69)
Insulin	42 (9.25)

## Discussion

The aim of pharmacological therapy in the treatment of osteoporosis is to prevent fractures. The Fracture Intervention Trial (FIT) Research Group in a randomized, double-blind, placebo-controlled trial consisting of 2,027 women with at least one vertebral fracture tested the hypothesis that alendronate decreases the risk of new vertebral fracture during a 3-year follow-up. 32 The risk of suffering from a further vertebral fracture was 8% in the alendronate group and 15% in the placebo group. The risk of other fracture was also lower in the alendronate group (13.6 vs. 18.2%). 32 Another study comprising of 4,432 postmenopausal women from the FIT tested the hypothesis that taking alendronate for 4 years would decrease the risk of vertebral fractures in women who have low BMD but no vertebral fractures. 33 There was a 36% reduction in vertebral fractures in the alendronate group, and it was reported that alendronic acid increased BMD at all sites as measured by dual X-ray absorptiometry. 33 In another controlled, double-blind, multicenter study from FIT found that alendronate is effective in lowering the risk of vertebral fractures in women with T scores of −1.6 to −2.5 (i.e., do not meet the BMD criterion for osteoporosis). 34 This study consisted of 3,737 postmenopausal women, with the risk of vertebral fracture being significant lower in the alendronate group

Despite good evidence that pharmacological therapy for osteoporosis reduces the risk of fragility fractures, unfortunately, several studies reported a low rate of initiation of osteoporotic treatment following a hip fracture. Jennings et al 35 conducted a study comprising of 51,346 patients older than 64 years from 318 hospitals in the United States suffering from a fragility fracture of the hip between October 2003 and September 2005. Calcium and vitamin D were prescribed to 6.6% ( *n* = 3,405) of the patients, while antiresorptive or bone-forming drugs were given to 7.3% ( *n* = 3,763) of patients. However, only 2% ( *n* = 1,023) received the ideal treatment (i.e., calcium and vitamin D together with an antiresorptive or bone-forming drug). Gonnelli et al 12 performed a retrospective study on 697 patients from four centers in Italy (Siena, Verona, Naples, and Palermo) with a low-energy hip fracture during the year 2014. Calcium and vitamin D were started in 18.8% of patients, while only 9.7% of patients were started on antiresorptive or bone-forming medications.

Cuschieri et al 36 conducted a retrospective study on all patients over the age of 60 years who suffered from a low-energy hip fracture in Malta in the year 2011. None of the patients were prescribed calcium, vitamin D, or bisphosphonates during their stay in the acute hospital. Further fractures of the hip within a year occurred in 6% of the individuals, with the authors highlighting the financial burden of these refractures. These results are similar to our study where only one patient was started on treatment during the stay in the acute hospital, and on discharge from rehabilitation hospital, only 2.6% of patients were prescribed osteoporosis medications.

For osteoporosis treatment to work, patients need to be compliant with treatment. Huybrechts et al 37 in their study found that three-quarters of the patients had medium to poor compliance with osteoporosis treatment. Low compliance was associated with a 37% higher risk of hospitalization and a 17% increase in fracture rate. Rabenda et al 38 conducted a study on all postmenopausal women suffering from a hip fracture between April 2001 and June 2001 in the entire Belgian population. This consisted of 23,146 individuals. Only 6% received pharmacologic osteoporosis treatment (4.6% given alendronate, 0.7% given risedronate, and 0.7% given raloxifene). At 6 months, 40% stopped taking alendronate, while at 1 year only 41% of those prescribed alendronate were compliant.

Once-yearly regimen with intravenous bisphosphonates that do not require individuals to remain standing and are easier to tolerate might improve compliance. Lyles et al 39 in a randomized, double-blind, placebo-controlled trial found that a yearly zoledronic acid infusion within 3 months following a hip fracture was associated with lower risk of new fractures and decreased mortality. In this trial, 1,065 individuals received 5 mg intravenous zoledronic acid yearly, while 1,062 individuals received a placebo. The rates of new fragility fractures were 13.9% in the placebo group and 8.6% in the zoledronic acid group (i.e., a 5.3% absolute risk reduction and a 35% relative reduction). Mortality rate in the placebo group was 13.1%, while in the zoledronic acid group it was 9.6% (i.e., 28% reduction in mortality). There were no reported jaw osteonecrosis or problems with fracture healing in this trial.

The findings of our study must be seen in light of some limitations. Data was collected retrospectively, assuming the drug history on the discharge letter was correct. We suggest the issuing of local guidelines on the prescription of antiosteoporosis medication following fragility fractures. Following this, prospective audits should be performed ensuring adherence to the guidelines and patients' compliance to medications. Unfortunately, despite the Maltese Islands having a free national healthcare system, antiosteoporosis medications are not on the free medication entitlement scheme; thus, anti-osteoporosis medications should be prescribed routinely following low-energy hip fractures, and this will negatively affect compliance.

## Conclusion

Osteoporosis is a bone disease that is both preventable and treatable. Unfortunately, most studies worldwide show that only a small percentage of those at increased risk of fracture are assessed and treated, even following a fragility fracture. The rate of osteoporosis treatment in Malta is no different with only 2.64% of the studied population receiving osteoporosis therapy. Knowing that fragility fractures are of a great social and financial burden with a high morbidity and mortality rate, it is important that this issue be addressed. An active role of the orthogeriatrics team in the management and treatment of osteoporosis might improve treatment rate and decrease refracture and mortality rates. There should be increased osteoporosis awareness in Malta and a national BMD screening program should be set up.
